# Development and Psychometric Evaluation of the TOGETHER Family‐Reported Experience Measure of Nurse‐Facilitated Family Engagement in Adult Acute Care

**DOI:** 10.1111/hex.70699

**Published:** 2026-05-28

**Authors:** Julie Cussen, Michael J. Ireland, Georgia Tobiano, Amanda Corley, Anne M. Eskes, Whitney Kelly, Lachlan Kelly, Jolanda M. Maaskant, Benjamin R. Mackie, Thom J. T. Westdorp, Andrea P. Marshall

**Affiliations:** ^1^ Gold Coast University Hospital, Gold Coast Hospital and Health Service Gold Coast Australia; ^2^ School of Nursing and Midwifery Griffith University Gold Coast Australia; ^3^ Faculty of Sciences University of Southern Queensland Ipswich Australia; ^4^ Division of Surgery Princess Alexandra Hospital Brisbane Australia; ^5^ Department of Surgery Amsterdam UMC, University of Amsterdam, Amsterdam Public Health Amsterdam the Netherlands; ^6^ Young Allies Foundation LTD Brisbane Australia; ^7^ Department of Internal Medicine, Amsterdam UMC University of Amsterdam Amsterdam the Netherlands

**Keywords:** acute care, family engagement, person‐centred care, scale development

## Abstract

**Background:**

Family engagement improves outcomes, shortens hospital stays, and enhances patient and family experiences, yet remains inconsistently enacted despite nurses' pivotal role. Existing instruments focus on paediatrics or critical care, measure activation rather than enacted engagement, or lack robust psychometric evidence. No family‐reported measure captures nurse‐facilitated engagement in general adult acute care.

**Objective:**

To develop and psychometrically evaluate a family‐reported experience measure of nurse‐facilitated family engagement in adult acute care.

**Methods:**

A two‐phase mixed‐methods instrument development was completed. Phase 1 involved construct definition, item generation, expert review (content validity), and cognitive interviews. Phase 2 used a cross‐sectional survey (*n* = 237) to test factor structure, reliability, and construct (convergent and discriminant) validity.

**Results:**

TOGETHER (TOwards Gathering Evidence to evaluaTe in‐Hospital family partnERships) is a 23‐item scale. Structural analysis of the 20‐item analytic core (three context‐contingent items retained for content coverage but excluded a priori from factor modelling) supported a bifactor model comprising a general factor (‘family partnerships’) and two specific factors (‘communication’, ‘collaboration’). Model fit was good (CFI = 0.992, RMSEA = 0.060), and total‐score reliability was adequate (*ω* = 0.79). Specific factors were moderately correlated (*r* = 0.61).

**Conclusion:**

TOGETHER provides a total score for evaluation and monitoring of nurse‐facilitated family engagement in adult acute care; domain‐level patterns may be examined descriptively but lack sufficient reliability for standalone scoring. The present evidence applies to the English‐language version only. Cross‐site benchmarking, cross‐group comparison, and cross‐linguistic extension require further validation.

**Patient or Public Contribution:**

Family caregivers with lived experience collaborated as study investigators throughout the study. They contributed lived healthcare and caregiving experience as well as specialist expertise in person‐centred care policy. Their involvement spanned study conduct, item development and manuscript preparation, including review of the final manuscript prior to submission. An additional group of people with lived experience provided their perspectives on the instrument during pretesting.

**Abbreviations**
ω: omega total reliability
ωh: omega hierarchical
ωhs: omega hierarchical subscale
AIC: Akaike information criterion
AVE: average variance extracted
BIC: Bayesian information criterion
CFA: confirmatory factor analysis
CFI: comparative fit index
COSMIN: COnsensus‐based Standards for the selection of health Measurement Instruments
CVI: content validity index
ECV: explained common variance
EFA: exploratory factor analysis
EORTC: European Organisation for Research and Treatment of Cancer
FAME: family engagement measure
FAM‐Activate: family activation measure
FCC: family‐centred care
FICQ: family involvement in care questionnaire
FIML: full information maximum likelihood
GRIPP2: Guidance for Reporting Involvement of Patients and the Public version 2
I‐CVI: item‐level content validity index
KMO: Kaiser–Meyer–Olkin
MAR: missing at random
MCAR: missing completely at random
MLR: robust maximum likelihood
MNAR: missing not at random
PCC: person‐centred care
PRO: patient reported outcome
PUC: percentage of uncontaminated correlations
RMSEA: root mean square error of approximation
SABIC: sample‐size‐adjusted Bayesian information criterion
S‐CVI/Ave: scale‐level content validity index (average)
SRMR: standardised root mean square residual
TLI: Tucker–Lewis index
TOGETHER: TOwards Gathering Evidence to evaluaTe in‐Hospital family partnERships
VET: vocational education and training
WLSMV: weighted least squares mean and variance adjusted

## 1 Introduction

Family engagement is an intentional partnership among clinicians, patients and families, sharing information and decisions to support patient autonomy [1]. The healthcare team recognises families as meaningful contributors to care [2], while keeping the patient's preferences and values central [3]. Family engagement is a foundational component of person‐centred care (PCC), an approach characterised by holistic perspectives and recognition of individuality [4].

Family engagement is increasingly recognised as essential for safe, high‐quality care [5], improving treatment adherence [6], self‐management [7], satisfaction and trust [7] and reducing complications [8]. For families, it improves situational understanding, communication with clinicians, and feeling valued by the healthcare team [5, 7]. For patients and families, it builds empowerment while reducing stress, anxiety and depression [9], and for health systems, it is associated with shorter hospital stays and fewer readmissions [10].

Despite these benefits, family engagement remains underutilised in adult acute care settings. Nurses provide continuous bedside care and are families' primary point of contact, placing them at the centre of engagement [11]. However, practice varies despite generally positive attitudes toward family involvement [12]. Factors include resistance to changing traditional practices and inconsistent willingness to engage families [13]. Families express a strong desire to be partners in care, but report clinicians rarely assess their capacity or willingness to participate [14]. Barriers also include unclear staff responsibilities and discharge planning that overlooks family dynamics [14]. Acute care is fast‐paced, with rapidly changing conditions and competing clinical priorities [12], creating constraints that foster a ‘task‐ and time‐driven’ culture [15], at odds with meaningful engagement. In dynamic, high‐acuity [16] episodes of care, family needs are viewed as secondary [17], particularly when patients can self‐advocate and may not need substitute decision‐makers, further limiting engagement.

Improving family engagement in acute care requires methods that capture how engagement is enacted at the bedside, including the ways nurses facilitate or hinder families' participation. A practical approach is to use a family‐reported measure that assesses specific engagement processes, such as how nurses enable families to participate meaningfully in patient care. Hospitals routinely collect patient experience data [18], but the instruments used rarely assess family engagement. Existing family experience measures tend to focus on overall satisfaction or communication quality [19], rather than the relational practices through which nurses enable engagement. Consequently, validated instruments that capture family‐reported experiences of nurse‐facilitated engagement in acute care are lacking.

Existing family engagement instruments were developed for paediatric contexts [2] or in adults, for critical care or acute cardiac settings [20]. Examples include the Family Engagement (FAME) instrument [21], the Family Activation Measure (FAM‐Activate) [22], the Family Involvement in Care Questionnaire (FICQ) [23], and a family readiness measure for early mobilisation [24]. Across these tools, psychometric evaluation is mixed, and the focus is often on family activation or context‐specific practices rather than the family's experience of nurse‐facilitated engagement in general acute care. As a result, the targeted and context‐bound nature of these tools constrains their generalisability to broader acute care settings, where heterogeneous patient populations present with varying acuity, unpredictable clinical courses and diverse engagement expectations. Moreover, although existing tools include items relevant to involvement, none capture family‐reported experiences of nurse‐facilitated engagement. Several assess adjacent constructs, such as readiness to engage, rather than engagement as experienced during care [21, 22]. Even the most conceptually aligned measure was developed and content‐validated in a narrow surgical context, with key psychometric properties (e.g., reliability and broader validity evidence) not yet established [23].

Together, these limitations point to a need for a general context‐adaptable instrument that captures family‐reported experiences of nurse‐facilitated family engagement across acute care settings. Such a measure would support routine evaluation of practice, and, once measurement equivalence is established, benchmarking across units and services and targeted quality improvement. Accordingly, this study aimed to develop and psychometrically evaluate a family‐reported experience measure of nurse facilitation of family engagement in adult acute care.

## 2 Methods

**Figure 1:**
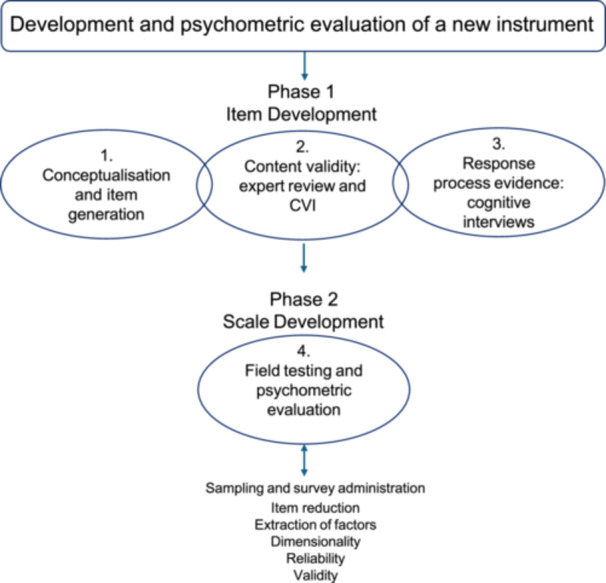
Two‐phase instrument development and psychometric evaluation process.

This study used a sequential mixed‐methods design. Figure 1 depicts the staged study process consistent with the approach described by Boateng et al. [24]

The research team was an international collaboration of six nurse investigators (three Australian, three from the Netherlands), and two consumer partners, bringing diverse healthcare and cultural perspectives. Phase 1 item development and content validity assessment were conducted collaboratively. The Australian and Dutch teams then pretested independently using country‐specific approaches, before reconvening to finalise instrument items based on shared findings. This article reports Australian data only.

In accordance with the Guidance for Reporting Involvement of Patients and the Public (GRIPP2) checklist [25], people with lived experience as family caregivers were involved as study investigators throughout this project, with reporting detailed in Supporting Information S1. An independent sample with lived experience, either as a patient or as a family member of a hospitalised patient, participated in instrument pretesting.

### 2.1 Phase 1 Item Development

#### 2.1.1 Conceptualisation

Guided by two complementary family engagement competency frameworks, the construct is defined as family members' experience of nurse‐facilitated family engagement in adult acute care, including nurses' communication and information‐sharing practices; their invitation and support for family participation in care and decision‐making; and respect for family preferences and needs (including cultural and spiritual needs) during admission. The dimensional structure was not sufficiently established to justify a single prespecified factor model [26]. Internal‐structure analyses, therefore, began with exploratory factor analysis (EFA) to identify plausible structural candidates, followed by structured model comparison within a confirmatory framework [26, 27].

#### 2.1.2 Conceptual Foundation

The instrument's theoretical foundation draws on two complementary family engagement frameworks [11, 28]. Hengeveld et al. [11] used multinational Q‐methodology to identify four hospital family‐centred care (FCC) themes: FCC preconditions; nurse‐patient‐family partnerships; FCC as fundamental to nursing; and positive attitudes to family involvement. Parmar et al. [28] developed a co‐designed clinician framework with six domains: recognising the caregiver role; communicating and partnering with caregivers; fostering resilience; navigating health and social systems and resources; and enhancing the culture and context of care. Together, these frameworks informed the nurse behaviours targeted in the instrument.

#### 2.1.3 Item Generation

An initial pool of 93 items was generated from the two frameworks [11, 28]. The research team iteratively reviewed items for clarity, relevance, redundancy, reading level and acceptability, reducing the pool to 28. Supporting Information S2 contains item screening, deletion rationale by category and readability procedures.

#### 2.1.4 Content Validity (Expert Review and CVI)

Eight content experts (independent of the research team) from Australia and the Netherlands rated item relevance on a scale from 1 = not relevant to 4 = very relevant [29]. Items with I‐CVI ≥ 0.78 were retained, and those < 0.78 were removed, eliminating four items [29]. The retained 24 items achieved S‐CVI/Ave = 0.93. Supporting Information S3 details the CVI procedures, item‐level ratings and synthesis of expert feedback.

#### 2.1.5 Response Process Evidence (Cognitive Interviewing)

Cognitive interviews were conducted with six health consumers. Items were assessed for comprehension and response option functioning using think‐aloud procedures [30, 31]. Revisions included removing one repetitive item, reordering for cognitive flow and changing from agreement to frequency response options. This phase resulted in the final 23‐item instrument. Supporting Information S4 provides the full protocol.

### 2.2 Phase 2: Field Testing and Psychometric Evaluation

#### 2.2.1 Study Design and Psychometric Analyses

A cross‐sectional survey design provided data for item analysis, item reduction, EFA, internal consistency and construct validity checks [24].

#### 2.2.2 Study Setting

Data were collected on adult inpatient units at two metropolitan Australian hospitals. Acute care was defined as general medical or surgical inpatient units. Inpatient unit specialities are listed in Supporting Information S5.

#### 2.2.3 Sampling and Survey Administration

Eligible participants were family members aged ≥ 18 years of adult inpatients, expected to visit at least twice weekly. ‘Family member’ was defined broadly as any person important to the patient, irrespective of legal or biological relationships (e.g., siblings, friends and neighbours) [32]. English verbal and written proficiency was required due to limited translation capacity [32].

On data collection days, research assistants screened ward patients and confirmed eligibility with the unit team leader or clinical facilitator. Eligible family members were approached and provided a written and verbal study description. Surveys were administered in paper format. Participants could complete the survey independently (with collection at an agreed time) or with the research assistant who recorded responses.

Sample size planning aimed for ≥ 10 participants per item with a minimum *N* = 200, noting that EFA sample adequacy depends on factor loadings, indicators per factor, and model error rather than ratios alone [33]. We therefore targeted *N* ≥ 230 for the 23‐item pool, meeting the 10:1 guideline [34] while monitoring item correlations and factor stability during analysis [35].

### 2.3 Data Collection

#### 2.3.1 Demographic Information

Participants reported age, gender, cultural or ethnic identification, relationship to the patient, education, employment, self‐rated health and languages spoken other than English.

#### 2.3.2 The TOGETHER Scale

Participants completed the 23‐item TOGETHER scale with reference to the current admission and inpatient unit only. Items assessed nurse‐facilitated engagement behaviours and related experiences. Questionnaires were entered into Excel by the lead investigator. The full item set is in Supporting Information S6.

#### 2.3.3 Data Analysis

Internal‐structure evidence was examined using a staged factor‐analytic approach [36]: EFA to explore dimensionality, followed by structured model comparison within a CFA framework using WLSMV [27]. Descriptive analyses were conducted in JASP v0.95.3 [37], with psychometric analyses in Mplus v8.11 [38], and R v4.4 [39].

##### 2.3.3.1 Step 1: Assessing Data Suitability for Factor Analysis

Missing data patterns were examined at both respondent and item levels before structural modelling, because respondent‐level any‐missingness is not equivalent to cell‐level missingness in the covariance matrix used for analysis [40, 41]. High any‐missingness across the full 23‐item survey was concentrated in three context‐contingent items (Q21, cultural needs, 70%; Q22, religious/spiritual needs, 73%; Q23, discharge information, 41%) that were excluded a priori from all factor models. For the remaining 20‐items, cell‐level missingness was approximately 7%, with most items exceeding 85% completion. Little's MCAR test indicated that missingness was not completely random, *χ* ^2^ (1207) = 1451.07, *p* < 0.001 [40], but a significant result does not establish MAR or diagnose MNAR [40]. Given the pattern of nonresponse, MAR was treated as a defensible working assumption. The main analyses used WLSMV with pairwise‐present data [42], and robustness was examined using MLR with FIML [43].

Items Q21–Q23 should be treated cautiously; they provide conceptual coverage but do not have the psychometric evidence in this dataset. All subsequent factor analyses proceeded on the 20‐item set. Item‐level descriptive statistics for the 20 analysed items, including mean (M), standard deviation (SD), skewness, kurtosis and completion rates, are reported in Supporting Information S7.

##### 2.3.3.2 Step 2: Factor Extraction Method

All primary factor analyses used WLSMV, which estimates polychoric correlations with pairwise‐present data and is appropriate for ordered categorical data [42, 44]. A planned sensitivity analysis using MLR with FIML treated the five‐category responses as approximately continuous. This analysis served only as a robustness check, not as a basis for primary structural inference, because MLR can produce biased fit indices and standard errors with ordinal data even if structural parameters are recovered adequately [45]. Consistency across estimators is reported as robustness evidence, but all primary interpretations are based on WLSMV results.

##### 2.3.3.3 Step 3: Factor Retention and Model Fit Criteria

In EFA, we examined one to nine factor solutions using WLSMV, evaluating each for parsimony, loading pattern and interpretability. Factor‐retention decisions were informed by parallel analysis (Pearson and polychoric), Minimum Average Partial, Very Simple Structure, Optimal Coordinates, scree‐plot inspection and information criteria (AIC, BIC, sample‐size‐adjusted BIC). The EFA solutions showed substantial cross‐loadings and did not yield a stable independent‐clusters structure, consistent with a dominant general factor whose variance is redistributed across group factors under oblique rotation [26, 46].

We therefore prespecified two latent structures for comparison within a CFA framework: (a) A second‐order model, which constrains general‐factor influence to operate indirectly through lower‐order factors and imposes a proportionality constraint on loading ratios [47], and (b) a bifactor model, which relaxes these constraints by estimating each item's general and specific loadings freely. Both were estimated using WLSMV. In the bifactor model, the general factor was orthogonal to both specific factors, which were allowed to correlate. This comparison addressed not only model fit but whether the variance pattern supported total‐score versus subscale‐score defensibility [27, 48]. This represents structured model comparison within the development phase, not independent confirmatory validation; cross‐validation remains a priority.

Model fit was evaluated using *χ* ^2^, Root Mean Square Error of Approximation (RMSEA) with 90% CI, (< 0.08 often considered adequate; < 0.05 good), Comparative Fit Index (CFI)/Tucker Lewis Index (TLI), (≥ 0.90 acceptable; ≥ 0.95 good), and Standardised Root Mean Square Residual (SRMR (target ≤ 0.08) [49]. Because conventional cut‐offs are simulation‐contingent, fit was interpreted holistically, prioritising theoretical coherence and interpretability alongside statistical indices [49].

##### 2.3.3.4 Step 4: Rotational Method

Oblique rotation (Geomin) was used to support correlated factors, because dimensions of family engagement are expected to overlap rather than function separately. Oblique rotation accounts for the inherent correlations found in complex human behaviours [36].

##### 2.3.3.5 Step 5: Factor Interpretation, Scoring and Psychometric Criteria

Reliability was evaluated using omega (total *ω* ≥ 0.70). In bifactor models, general‐factor strength was indicated by omega hierarchical (ωh) ≥ 0.50 and Explained Common Variance (ECV) ≥ 0.60, while essential unidimensionality was supported by ωh ≥ 0.70 together with Percentage of Uncontaminated Variance (PUC) ≥ 0.80 and ECV ≥ 0.60. Specific‐factor uniqueness was examined using ωhs (≥ 0.30 to 0.50), and construct replicability using *H* ≥ 0.70 [27, 50].

Convergent validity was assessed via standardised loadings (≥ 0.50 acceptable; ≥ 0.70 strong) and AVE ≥ 0.50 [51]. Discriminant validity was examined via latent‐correlation 95% confidence intervals excluding 1.00 and √AVE exceeding interfactor correlations (Fornell–Larcker, interpreted cautiously), with ωhs ≥ 0.30 providing additional evidence of specific‐factor distinctiveness [52].

To support standardised implementation, practical scoring instructions, including handling of missing and ‘not applicable’ responses and minimum completion thresholds, are provided in Supporting Information S8.

## 3 Results

Between January and April 2025, 320 family members were approached; 64 (20%) declined. Surveys were distributed to 256 family members; 237 were returned (response rate 93%). Of the 23 items developed and content‐validated in Phase 1, 20 were submitted to structural (factor‐analytic) modelling. The three context‐contingent items (Q21, cultural needs; Q22, religious or spiritual needs; Q23, discharge information) were retained in the instrument for content coverage and equity but excluded a priori from factor modelling because high non‐response (70%, 73%, and 41%, respectively) reflected whether the experience was applicable to the family rather than item failure (see Section 2.3.3.1). Of the 237 respondents, 47 (20%) completed all 23 items; this 80% respondent‐level any‐missingness rate was driven largely by Q21–Q23. Across the 20‐item analytic set submitted to structural modelling, cell‐level missingness was approximately 7%, and most items had completion rates exceeding 85% (Table 1).

**Table 1:** Family member characteristics (*N* = 237).

Characteristic	*N* (%) or M (SD)
Age (years)	58.9 (15.7)
Gender	
• Female	184 (77.6)
• Male	53 (22.4)
Relationship to patient	
• Spouse/partner	115 (48.7)
• Child	65 (27.5)
• Other^a^	56 (23.7)
Ethnic/Cultural minority	
• No	195 (83.0)
• Yes	40 (17.0)
Education	
• Some high school	42 (17.7)
• Secondary school/VET	121 (51.1)
• University/Postgraduate degree	72 (30.6)
Employment status	
• Full‐time/Part‐time	107 (45.5)
• Retired	93 (39.6)
• Other^b^	37 (15.7)
Self‐reported health status	
• Excellent/Very good	148 (62.4)
• Good	61 (25.7)
• Fair	23 (9.7)
• Poor	5 (2.1)
Language other than English	
• Yes	35 (14.8)
• No	201 (85.2)

*Note:* The continuous variable (age) is reported as M (SD), denoting mean and standard deviation. All other variables are categorical and reported as *n* (valid %), where *n* is the count of respondents in that category and % is the proportion of valid (non‐missing) responses. Percentages may not total 100 due to rounding. Missing data: Relationship (*n* = 1), ethnicity (*n* = 2), education (*n* = 2), language (*n* = 1).

Abbreviation: VET = vocational education and training.

^a^ Other includes parent, sibling, friend, carer, and extended family member.

^b^ Other includes unemployed, student, homemaker, and unable to work.

### 3.1 Missing Data Patterns

Little's MCAR test indicated departures from strict MCAR, *χ* ^2^ (1207) = 1451.07, *p* < 0.001 [40]. At the item level, missingness was minimal for end‐of‐survey items (Q16–Q18, 0.4%–0.8%) and higher for items requiring specific care opportunities (e.g., Q3, 13%; Q10–Q11, 14%–16%; Q13, 13%).

### 3.2 Factorability

KMO indicated excellent factorability (0.941), and Bartlett's test supported factor extraction, *χ* ^2^ (190) = 2760.57, *p* < 0.001. The correlation matrix determinant was very small (3.331 × 10^−10^), indicating near‐singularity and possible multicollinearity among item clusters. Although no item pair exceeded *r* = 0.90 [51], groups of related items shared substantial covariance. Correlations were highest among Q6–Q11 and Q12–Q15, possibly reflecting context‐contingent co‐occurrence of care experiences rather than cleanly separable domains. Both the WLSMV and MLR models converged normally without warnings regarding non‐positive definite matrices or linear dependencies, and no Heywood cases were observed, confirming that the matrix was positive definite and computationally sufficient for estimation. The factor structure was therefore interpreted with caution regarding specific‐factor distinctiveness.

### 3.3 Factor Retention and Model Selection

Factor‐retention procedures did not converge on a single solution, and the EFA results (one‐to‐nine factor solutions) did not yield an interpretable simple structure, consistent with a higher‐order or bifactor structure.

### 3.4 Factor Solution

The second‐order model showed acceptable fit (CFI = 0.983, TLI = 0.981, SRMR = 0.064, RMSEA = 0.082 [0.072, 0.091]), but uniformly high higher‐order loadings (0.85–0.98) indicated that the lower‐order factors were heavily saturated by a dominant common source and only weakly differentiated. Under these conditions, the proportionality constraint imposed by the second‐order parameterisation may not reflect actual loading heterogeneity across items [47, 53]. The four‐factor EFA solution achieved acceptable fit but was not retained because substantial cross‐loadings (Q2–Q5, Q12–Q15, Q19) prevented clean item assignment.

In the bifactor model, each item loaded on the general factor and, where applicable, on one of two specific factors. The general factor was orthogonal to both specific factors, so that specific‐factor loadings represent residual domain‐specific covariance after accounting for the shared construct. The two specific factors were allowed to correlate. The model specified one general factor (‘family partnerships’, all items), two specific factors (S1 ‘communication’: Q1–Q5, Q16–Q20; S2 ‘collaboration’: Q12–Q15), with Q6–Q11 loading only on the general factor. This model achieved excellent fit (RMSEA = 0.060, CFI = 0.992, TLI = 0.990, SRMR = 0.037).

The bifactor model was preferred for three reasons: it provided a flexible representation of the covariance pattern without the proportionality constraint; it resolved cross‐loadings more transparently by modelling general and specific factors simultaneously with freely estimated loadings; and it allowed formal evaluation via bifactor‐specific indices (ωh, ωhs, ECV, PUC, H) of whether the general factor justified a total score and whether specific factors warranted subscale interpretation [27, 48, 50].

The bifactor model also provided a clearer separation of general and specific variance. As a robustness check, the candidate models were re‐estimated using MLR with FIML, treating item responses as approximately continuous. As expected, MLR loadings were marginally attenuated relative to WLSMV (largest difference < 0.03), but the structural pattern and model‐preference conclusions were unchanged. Absolute fit was somewhat poorer under MLR (RMSEA = 0.083 [0.073–0.093], CFI = 0.902, TLI = 0.880), consistent with known attenuation when ordinal indicators are treated as continuous [45]. Primary interpretation rests on the WLSMV analyses; the MLR results are reported only as evidence that conclusions were not estimator‐dependent. The two specific factors were correlated (*r* = 0.608, ~37% shared variance), indicating residual association between communication and collaboration after accounting for the general factor (Table 2).

**Table 2:** WLSMV standardised factor loadings for bifactor model.

Item	General factor (g)	Specific factor 1 (S1)	Specific factor 2 (S2)	*h* ^2^
S1 Items				
Q1	0.636	0.542	—	0.698
Q2	0.716	0.509	—	0.772
Q3	0.751	0.521	—	0.836
Q4	0.736	0.405	—	0.706
Q5	0.777	0.310	—	0.700
Q16	0.450	0.795	—	0.835
Q17	0.523	0.780	—	0.882
Q18	0.519	0.677	—	0.728
Q19	0.762	0.438	—	0.773
Q20	0.271	0.469	—	0.294
General only items				
Q6	0.892	—	—	0.796
Q7	0.935	—	—	0.874
Q8	0.884	—	—	0.782
Q9	0.901	—	—	0.812
Q10	0.948	—	—	0.899
Q11	0.959	—	—	0.920
S2 Items				
Q12	0.832	—	0.414	0.863
Q13	0.844	—	0.411	0.907
Q14	0.796	—	0.411	0.803
Q15	0.804	—	0.513	0.910

*Note:* A dash (—) indicates that the corresponding loading was fixed to zero because that item was not specified to load on that specific factor in the bifactor model. *h* ^2^ = communality (total variance explained).

### 3.5 Reliability

The bifactor indices informed score‐use recommendations. Total‐score reliability was adequate (*ω* = 0.79). ECV = 0.75 indicated that approximately three‐quarters of common variance was attributable to the general factor. However, ωh = 0.59 and PUC = 0.52 fell below benchmarks for essential unidimensionality (ωh ≥ 0.70, PUC ≥ 0.80) [27]; indicating the specific factors could not be ignored entirely. Critically, specific factors retained limited unique reliable variance (communication ωhs = 0.16; collaboration ωhs = 0.04), and subscale reliability was modest (ωs = 0.54 and 0.61). The total score is therefore the most defensible summary outcome; subscale scores should remain exploratory. This conclusion could not have been formally derived within the second‐order parameterisation, which does not yield ωh, ωhs, or ECV directly. The near‐singular correlation structure reinforced this interpretation: the combination of a small determinant, high general‐factor loadings, and low ωhs values converges on the same conclusion.

### 3.6 Convergent and Discriminant Validity

Convergent validity for the general factor was supported. Eighteen of the 20 items had high communalities (*h* ^2^ ≥ 0.70; mean *h* ^2^ = 0.79), and 15 items had strong general‐factor loadings (*λg* > 0.70). Items Q6–Q11 loaded very highly on the general factor (0.88–0.96), indicating strong representation of the core construct.

Specific factors showed varying patterns. Within S1 (‘communication’), Q16–Q17 showed strong specific loadings (0.78–0.80), reflecting respectful and easy‐to‐understand communication. The remaining S1 items showed weaker specificity; S2 (collaboration) showed modest but consistent specific loadings across items. Item Q20 (‘The nurses allow me to visit according to my wishes’) was the clearest item‐level psychometric concern (*h* ^2^ = 0.29, *λg* = 0.27), indicating weak alignment with the common‐factor structure. Q20 was provisionally retained to preserve coverage of visitation practices, but should be re‐evaluated in future samples with more heterogeneous visiting policies and revised or removed if weak performance persists.

In the bifactor model, the general factor ‘family partnerships’ was constrained to be orthogonal to the specific factors, so specific factor loadings reflect domain‐specific covariance after accounting for the general tendency. The two specific factors were allowed to correlate and showed a substantial association (*r* = 0.61), indicating related but distinguishable domains. Discriminant validity was supported: √AVE for S1 = 0.73 and √AVE for S2 = 0.78 exceeded the S1–S2 correlation = 0.61, and the 95% CI for the S1–S2 correlation (0.48–0.73) excluded 1.00.

## 4 Discussion

This study addressed a gap in healthcare measurement by developing and providing initial psychometric evidence for a family‐reported experience measure of nurse‐facilitated family engagement in adult acute care. The instrument yields a reliable total score for evaluating practice and monitoring change within comparable populations; cross‐group or cross‐site comparisons should be deferred until measurement invariance is established. Factor modelling indicated a strong general factor, making the total score the most defensible summary outcome for routine use. Two sub‐dimensions, communication and collaboration, may be reported descriptively or used in exploratory analyses, but their limited reliability means they should not be used as stand‐alone outcomes for confirmatory hypothesis testing, clinical decision‐making or between‐group comparisons. Where the domain‐specific signal is clearest, for example, items Q16–Q17 within communication, item‐level reporting or descriptive summaries may be informative, but routine confirmatory use of subscale scores is not yet warranted.

Question 20 (‘The nurses allow me to visit according to my wishes’) emerged as the clearest item‐level psychometric concern in the present study. Its low communality (*h* ^2^ = 0.29) and weak general‐factor loading (*λg* = 0.27) indicate that it is not well explained by the common‐factor structure and should not presently be treated as an equally well‐supported indicator of nurse‐facilitated engagement. This may reflect ambiguous wording or interpretation, restricted variability (e.g., uniformly permissive or uniformly constrained visiting), or the possibility that visiting arrangements are driven more by ward policy and clinical status than by nurses' facilitation [54]. Previous research using the FAME tool found family visitation consistently scored highest among engagement domains [21, 55], suggesting the present finding may reflect item wording or construct misalignment rather than visitation being unimportant to families. Alternatively, visiting arrangements may represent a related but distinct aspect of experience that sits outside nurse‐facilitated engagement as operationalised in the TOGETHER scale. The visiting policy at the study sites was restricted between the hours of 10:00 am and 8:00 pm. While visitation matters to families, it may primarily reflect organisational policies and system‐level constraints beyond nurse control, whereas other items in the instrument capture nurse‐family interactions that nurses can directly influence. This structural difference may help explain why Q20 fails to load strongly with behaviours under direct nurse control. Accordingly, Q20 has been retained provisionally, supported by a supplementary sensitivity analysis confirming that its removal did not alter model fit or the remaining loading pattern, demonstrating that its inclusion does not degrade the instrument. Nevertheless, it remains subject to re‐evaluation: if it continues to show *h* ^2^ < 0.40 and *λg* < 0.40 in future samples from services with heterogeneous visiting policies, it should be removed or reconceptualised as a separate system‐level indicator. The very small determinant of the correlation matrix indicates that the lower‐order groupings should not be interpreted as strongly independent dimensions. In acute‐care contexts, engagement opportunities often occur in bundles: when a family is invited into planning and shared decision‐making, several partnership‐related items (Q6–Q11, Q12–Q15) may be endorsed together; when those opportunities do not arise, the same items cluster at uniformly lower endorsement or higher missingness. Carman et al.'s framework conceptualises engagement as a progression from simple information exchange to deeper partnership [3], and the TOGETHER item clusters map onto this continuum: Q6–Q11 capture foundational nurse behaviours, while Q12–Q15 capture collaborative planning that presupposes earlier opportunities. Context‐contingent co‐occurrence can inflate inter‐item correlations and contribute to residual groupings that reflect shared exposure to clinical events as much as distinct latent constructs. This interpretation is consistent with the low ωhs values (communication = 0.16; collaboration = 0.04) and supports treating the total score as more defensible than the subscale scores.

The bifactor model was preferred not on the basis of superior fit alone, but because it best captured this covariance pattern and most directly addressed the central scoring question: whether a total score is defensible and whether subscale scores carry enough unique signal to warrant interpretation [27, 48, 50]. Future validation should re‐examine whether the communication and collaboration groupings stabilise as stronger specific factors in larger and more diverse samples.

Progression along the engagement continuum requires nurse facilitation [56]. Families are unlikely to experience partnership‐level practices unless they first encounter foundational opportunities for involvement. When organisational pressures such as time constraints [12, 13], staffing [57] and ward cultures [15] limit nurses' capacity to support engagement [58], families may not progress to higher‐level engagement practices [58]. This helps explain why items Q6–Q11 and Q12–Q15 tended to move together, with uniformly low or high ratings, reflecting a cumulative sequence of engagement opportunities rather than conceptual duplication.

The missing data profile is consistent with the same interpretation. Items that presume partnership‐level opportunities, such as joint care planning and goal setting (Q13, 13% missing), were more often left unanswered, consistent with these experiences not occurring for some participants. Some non‐response may also reflect response burden during acute hospitalisation, where stress, uncertainty, fatigue and competing demands [59] can make items that bundle together multiple ideas harder to answer [59] (e.g., Q11 combines comfort, involvement and decision‐making). Additionally, some items may feel relationally sensitive with respondents reluctant to provide critical feedback during an ongoing admission [59, 60], as noted in informal participant comments about visiting according to their wishes (Q20). The three context‐contingent items (cultural needs, Q21; religious or spiritual needs, Q22; discharge information, Q23) showed substantial structural non‐response, consistent with important domains that are not universally relevant or not yet encountered during admission.

Together, these patterns suggest that inter‐item clustering and non‐response are shaped mainly by whether families had the opportunity to experience higher‐level engagement, and when the survey was administered, rather than by item redundancy. This supports the TOGETHER scale's design as a bedside measure of enacted nurse facilitation, while highlighting practical implementation considerations: administer after families have had a realistic chance to observe planning and decision‐making processes, provide clear instructions about the existing ‘not applicable’ option, and pre‐specify how such responses will be handled analytically as context‐dependent missingness.

Despite weaker factor‐model performance, the discharge planning item (Q23) warrants retention because it captures a high‐impact, practice‐relevant aspect of engagement. Timely engagement in discharge planning is critical not only for patient and family experience but also for optimising acute care resources, considering up to one in 10 public hospital beds in Australia are currently occupied by patients who are medically ready for discharge but awaiting appropriate care settings [61]. Families consistently report wanting to be involved in discharge planning [62, 63], and collaborative discharge discussions can reduce anxiety, improve preparedness for caregiving [62] and increase family satisfaction [56]. Early engagement can also help activate necessary services and reduce delays, supporting smoother transitions and better care outcomes. Yet engagement in discharge planning remains difficult to deliver in practice, particularly when time or skill constraints limit meaningful collaboration [56]. Retaining Q23 preserves coverage of a domain that is central to quality improvement in acute care.

Similarly, cultural and religious preference items warrant retention for content coverage and equity. Australian health services operate in a highly diverse context [64], and person‐centred engagement includes recognising and responding to cultural, religious, and spiritual needs when they are present. Although these needs were not salient for many participants in this sample, the items capture important experiences for those who do hold such preferences, and align with evidence that clinicians' integration of cultural and spiritual needs is variable and influenced by training and organisational support [65, 66].

In early‐stage instrument development, item retention decisions require balancing internal‐structure evidence against construct coverage [67, 68]. Accordingly, Q21–Q23 were retained on the basis of Phase 1 content validity evidence despite not being structurally tested in this dataset, while Q20 showed weak structural performance and requires re‐evaluation. These items should therefore be reported separately from the TOGETHER total score as supplementary descriptive indicators rather than included in core scoring. This approach is consistent with established patient‐reported outcome (PRO) practice (e.g., EORTC QLQ‐C30) [69].

Completion rates for these items may also provide useful service level information: a high proportion of ‘not applicable’ responses may signal that relevant care processes are not routinely offered rather than that families do not value them. Future evaluation will require targeted sampling from settings where these experiences are more consistently applicable, including culturally and linguistically diverse catchments and units with structured discharge‐planning and spiritual‐care provision.

### 4.1 Strengths and Limitations

Sustained consumer partnership from early item development through manuscript review ensured experiential knowledge informed the entire project. The final items, therefore, reflect family experiences in acute care, rather than solely clinical or theoretical assumptions, supporting real‐world relevance. Grounding item generation in existing frameworks strengthens the instrument's conceptual basis. The two‐site design within one health service captured family perspectives across different ward contexts, improving representativeness, while collaboration with investigators in the Netherlands also supported development and may enhance transferability beyond Australia.

Several limitations warrant consideration. Limited ethnic diversity, likely reflecting hospital catchments, and exclusion of non‐English speaking family members may have reduced variability and introduced selection bias. Families with limited English proficiency are more likely to experience communication barriers and reduced confidence navigating clinical environments [70], so their engagement experiences may differ from those of the families represented here. The high ‘not applicable’ rates for Q21 and Q22 may partly reflect this sample's limited diversity. Psychometric evidence beyond the English‐language version remains unavailable because content, response‐process and structural evidence have not yet been established for other language groups. Validation in culturally and linguistically diverse samples remains necessary.

Further methodological limitations warrant acknowledgement. The near‐singular correlation structure suggests that specific‐factor distinctiveness may be partly sample‐dependent and requires confirmation in samples with more heterogeneous care trajectories. Measurement invariance was not tested; the COSMIN taxonomy treats this as a distinct property presupposing established internal structure [71, 72], and subgroup sizes in this study (*N* = 237 total) were insufficient for stable multigroup ordinal bifactor analysis, which requires approximately 200–300 per group [73, 74]. Until invariance is demonstrated, TOGETHER should be used for within‐group evaluation rather than cross‐group comparison or cross‐site benchmarking. The context‐contingent items Q21–Q23 will require targeted sampling in diverse catchments and in units with routine discharge‐planning and spiritual‐care provision.

### 4.2 Future Validation and Generalisability

The bifactor structure reported here is a candidate model identified within Phase 2 development and requires confirmation through independent confirmatory factor analysis in an a priori powered, independent sample before it can be considered established. This independent cross‐validation is the immediate methodological priority. Beyond replication, future validation studies should sample deliberately across care contexts, hospital types, unit specialities, acuity profiles, and staffing models to determine whether the observed structure reflects stable dimensions of nurse‐facilitated engagement, or whether it is partly an artefact of setting‐specific workflows. Once a broader structural base is established, measurement invariance should be tested across gender, hospital type, unit speciality and cultural background, with per‐group sample sizes adequate for ordinal bifactor specifications, as a prerequisite for cross‐group comparison and cross‐site benchmarking [74]. Importantly, some of the patterns seen in this study, such as strong correlations between items related to partnership, may not solely reflect the underlying concept being measured. Instead, they could also be influenced by participants having experienced the same or similar clinical events. For example, when a family is invited into planning and shared decision‐making, multiple partnership‐level items may be endorsed together; when those opportunities do not arise, those same items may cluster at uniformly low endorsements or higher missingness. This co‐occurrence can inflate inter‐item correlations and contribute to near‐singular correlation matrices, creating apparent ‘factors’ that reflect shared exposure to events (ward rounds, goal‐setting conversations, discharge planning) rather than distinct latent constructs.

To address this future studies should, (1) recruit across units with different care trajectories and predictable versus unpredictable admissions, (2) record timing and exposure indicators (day of admission, proximity to rounds, discharge status, family presence frequency, whether planning meetings occurred), and (3) test measurement invariance across these context strata, and where feasible, across demographic and hospital groups. Designs that include repeated administration across the admission would also clarify how engagement changes over time and allow responsiveness testing, including whether partnership‐level experiences emerge later than foundational involvement practices. Future validation should prioritise formal translation and cultural adaptation following established methodology [75, 76], with subsequent testing of structural validity and measurement invariance across language groups before multilingual administration is undertaken. The existing Australian Dutch collaboration provides immediate infrastructure for the first cross‐linguistic validation.

### 4.3 Clinical Implications

The timing of TOGETHER scale administration should be considered based on measurement intent and ward‐specific length‐of‐stay patterns. Early administration captures foundational engagement behaviours but may miss partnership‐level practices, while delayed administration risks missing families due to rapid discharge. Importantly, the scale can function as both an evaluative and an improvement tool. By collecting and feeding back unit‐level scores, clinical teams can identify where engagement practices fall short of partnership ideals and target quality improvement initiatives accordingly. Regular measurement enables iterative testing of strategies to move beyond task‐oriented interactions towards meaningful partnership.

## 5 Conclusions

The TOGETHER survey is a novel, evidence‐based instrument for assessing family‐reported experiences of nurse‐facilitated family engagement in adult acute care. Developed through a rigorous two‐phase process with sustained consumer involvement, the 23‐item scale demonstrated strong content validity (S‐CVI/Ave = 0.93) and good structural validity, with a bifactor model providing the most interpretable representation of the data. The instrument yields a reliable total score (*ω* = 0.79) supported by a dominant general factor, making the total score the most defensible summary outcome for routine evaluation and monitoring. Convergent and discriminant validity indicators were also supportive.

The present structural and reliability evidence applies to the English‐language version administered to English‐speaking family members in Australian acute‐care settings, and most directly to the 20‐item analysed core. Q20 (visitation) and the context‐contingent items Q21–Q23 remain provisional and require targeted evaluation before they can be considered psychometrically established components of the scored instrument. Further validation across diverse populations, settings and staffing models is recommended to confirm structural stability, establish measurement invariance and strengthen guidance on subscale and context‐contingent item use. Until invariance is demonstrated, TOGETHER is best suited for within‐context evaluation and monitoring rather than formal cross‐group or cross‐site benchmarking. Extension to non‐English‐speaking populations will require formal translation, cultural adaptation and cross‐cultural validation.

## Author Contributions

**Julie Cussen:** conceptualisation, data curation, formal analysis, funding acquisition, investigation, methodology, project administration, resources, validation, visualisation, writing – original draft, writing – review and editing. **Michael J. Ireland:** formal analysis, software, methodology, supervision, validation, writing – review and editing. **Georgia Tobiano:** conceptualisation, data curation, formal analysis, investigation, methodology, resources, validation, writing – review and editing, funding acquisition, supervision, visualisation. **Amanda Corley:** supervision, validation, visualisation, writing – review and editing. **Anne M. Eskes:** conceptualisation, data curation, writing – review and editing. **Whitney Kelly:** conceptualisation, data curation, writing – review and editing. **Lachlan Kelly:** conceptualisation, data curation, writing – review and editing. **Jolanda M. Maaskant:** conceptualisation, data curation, writing – review and editing. **Benjamin R. Mackie:** supervision, validation, visualisation, writing – review and editing. **Thom J.T. Westdorp:** conceptualisation, data curation, writing – review and editing. **Andrea P. Marshall:** conceptualisation, data curation, formal analysis, funding acquisition, methodology, supervision, validation, visualisation, writing – review and editing.

## Ethics Statement

All phases of the research had human research ethics approval.

## Consent

All participants were provided verbal and written ethics‐approved information, and the return of the survey implied consent.

## Conflicts of Interest

The authors declare no conflicts of interest.

## Supporting information

Supporting File

## Data Availability

The data that support the findings of this study are available from the corresponding author upon reasonable request and upon consideration of any ethical/privacy concerns.
