# Supplementary material for: Development and Psychometric Evaluation of the TOGETHER Family‐Reported Experience Measure of Nurse‐Facilitated Family Engagement in Adult Acute Care
Source: Health Expect. 2026 May 28;29(3):e70699. doi: 10.1111/hex.70699 (PMC13240197; doi:10.1111/hex.70699)
Supplement: Supplementary file 1 — Supporting File [file HEX-29-e70699-s001.docx]

# Supporting File S1

Consumer engagement GRIPP2 short form ^1^

| Selection and topic | Item |
| --- | --- |
| 1: Aim | To develop consensus and actively involve consumers as research partners in the development of a new instrument measuring family engagement in acute care settings, ensuring that items reflect authentic family experiences. |
| 2: Methods | Two consumers were invited to join the research team, one based in England and one in Australia, to contribute to item development and the consensus process. They participated in discussions regarding refining individual items, reviewed comprehension, and provided suggestions for revisions, deletions, and amalgamations. Feedback was provided from a lay perspective. Consumers also provided ongoing feedback via study updates and contributed to the manuscript through review prior to submission. |
| 3: Study results | Consumer partners contributed to the study by:   - Reviewing initial items found in the literature and discussing their relevance - Drawing on their wider lived experience to highlight the importance of including items with clear, inclusive lay- language appropriate for the target population - Providing feedback on item comprehension from a lay perspective, including comments on previous iterations - Contributed to manuscript review, ensuring that the reporting reflected the intended meaning and relevance for families |
| 4: Discussions and conclusions | Consumer involvement influenced important aspects of the study. Their lived experience of an unwell family member provided authentic perspectives that enhanced the relevance and appropriateness of the instrument items. Early engagement in item development allowed consumers to shape content based on experiential knowledge, ensuring items reflected real world family experiences rather than clinical or theoretical constructs.  A digital meeting format (TEAMS) enabled flexible participation across international time zones while maintaining meaningful engagement. Multiple feedback rounds allowed for iterative refinement and demonstrated genuine incorporation of consumer input.  Limitations included the digital format potentially reduced the depth of discussion compared to face-to-face meetings, and the absence of formal research training for consumers which may have limited the technical detail of feedback on methodological aspects. Additionally, involving two consumers provided a relatively narrow range of family experiences.  For future studies earlier engagement in defining study scope, provision of brief research methods orientation, and inclusion of a more diverse range of family experiences could further enhance the impact of consumer involvement. |
| 5: Reflections/critical perspective | The iterative feedback model supported meaningful consumer input during early study phases and allowed contributors to see how their perspectives informed instrument development, which helped sustain engagement.  Digital participation enabled flexible involvement despite geographic differences and work or travel commitments.  A key challenge was sustaining consumer engagement over the lengthy research process. Despite regular study updates via newsletters and invitations to participate in manuscript review along the way, long-term involvement can be difficult to maintain.  Balancing instrument development procedures with meaningful consumer input was also challenging. Predetermined frameworks may have limited opportunities for consumers to introduce concepts outside of the study’s initial scope, and the intervals between feedback cycles, although not rushed, may have limited opportunities for deeper reflection.  For future studies, earlier consumer involvement in defining the constructs to be measured, rather than refining pre-determined items, could support and further enhance engagement. |

Reference

1. Staniszewska S, Brett J, Simera I, Seers K, Mockford C, Goodlad S, et al. GRIPP2 reporting checklists: tools to improve reporting of patient and public involvement in research. bmj. 2017;358.

# Supporting File S2

Item generation

An initial pool of 93 items was generated from the two theoretical frameworks.^1, 2^ Hengeveld et al., contributed 72 competency items for family centered care in hospital settings,^1^ while Parmar et al., contributed 21 items addressing clinician competencies for supporting family caregivers.^2^ Neither source represented a previously validated measurement instrument; rather both were competency frameworks developed through expert consensus methods. Hengeveld et al. used Q-methodology, while Parmar et al. used a stakeholder co-design approach to develop the competency domains and indicators, followed by a Modified Delphi process to validate the domain indicators. This approach ensured item generation was grounded in prior theory and empirical evidence, consistent with established instrument development procedures.^3,4^

The 93 items from both studies were compiled into an Excel spreadsheet to facilitate systematic review and comparison. Systematic reduction and refinement of the initial item pool was enacted through collaborative, iterative discussions among the study investigator team. These discussions incorporated multiple phases to ensure item clarity, precise operational definitions, and comprehensive coverage of content areas relevant to the family member target population.^5^ Team deliberations included critical evaluation of each items wording, structure, relevance, and clarity with consideration of potential participant interpretation.^6^ This process assessed overlap and redundancy among items ^7^ and ensured response categories were mutually exclusive and exhaustive. This included maintaining unidimensional item statements and adjusting wording for enhanced clarity.^6^ The process was guided by established criteria from Stone,^6^ and Hinkin,^7^ focusing on ensuring items were appropriate, intelligible, unbiased, unambiguous and inclusive. Readability was evaluated using the Flesch-Kincaid scale, with the goal of maintaining a reading level below Grade 8 to support accessibility across diverse health literacy levels.^8^

Our research team consisted of an international collaboration involved six nurse investigators (three from Australia, three from the Netherlands) and two consumer partners. The 93-item pool was reduced to 28 items through six investigator meetings attended by all nurse investigator team members. In parallel, the Australian team conducted three separate consultation rounds with consumers, incorporating their feedback into the investigator meeting discussions. Of 65 removals, 24 were redundant, 16 were out of scope, 13 failed clarity or unidimensional checks, 7 exceeded reading level targets after revision, and 5 were removed following consumer feedback on acceptability.

References

1. Hengeveld B, Maaskant JM, Lindeboom R, Marshall AP, Vermeulen H, Eskes AM. Nursing competencies for family‐centred care in the hospital setting: A multinational Q‐methodology study. Journal of advanced nursing. 2021;77(4):1783-99.

2. Parmar J, Anderson S, Duggleby W, Holroyd‐Leduc J, Pollard C, Brémault‐Phillips S. Developing person‐centred care competencies for the healthcare workforce to support family caregivers: Caregiver centred care. Health & Social Care in the Community. 2021;29(5):1327-38.

3. Cresswell J, Plano Clark V. Designing and conducting mixed methods research. 2nd edn Sage Publications Inc. Thousand Oaks, CA. 2011;6(2):1.

4. Devellis RF. Scale development: Theory and application. Los Angeles, CA: Sage Publications; 2012.

5. Morgado FFR, Meireles JFF, Neves CM, Amaral ACS, Ferreira MEC. Scale development: ten main limitations and recommendations to improve future research practices. Psicologia: Reflexão e Crítica : Psychology: Research and Review. 2018;30(1):1-20.

6. Stone D. Design a questionnaire. British Medical Journal. 1993;307(6914):1264-6.

7. Hinkin TR. A brief tutorial on the development of measures for use in survey questionnaires. Organizational research methods. 1998;1(1):104-21.

8. Jindal P, MacDermid JC. Assessing reading levels of health information: uses and limitations of flesch formula. Education for Health. 2017;30(1):84-8.

# Supporting file S3

Content validity

Content validation was conducted through consultation with an expert panel (n= 8),^1^ made up of nurse researchers and senior nurses in leadership positions. Five experts were based in Australia and three in the Netherlands. We perceived that the sample had content expertise because they have experienced, practiced or published papers on PCC or family engagement. The sampling strategy recruited experts from a different but related population to those used in the initial item development phase. All experts who were initially approached agreed to participate in content validation. Content experts completed content validity assessments via email.

Data collection was conducted over one round using a structured survey instrument containing explicit instructions regarding the assessment purpose, construct definition, and item evaluation guidance. The content validity index (CVI) was used to measure content validity. CVI can be measured at the item level (I-CVI) and at the scale level (S-CVI). For each item, experts rated relevance on a 4-point Likert scale. I-CVI represents the proportion of experts rating an item as 'quite relevant' or 'very relevant'. Items required an I-CVI of 0.78 or higher for retention.^1^

S-CVI was calculated by dividing the sum of all I-CVIs by the total number of items in the final scale. An S-CVI/Ave > 0.9 indicated excellent content validity.

Expert recommendations were analysed using a modified I-CVI approach where items scoring 1.0 were considered 'retain exactly'; items scoring > 0.78 indicated minor revision may be needed; and items scoring < 0.78 suggested major revision requirements.

Item clarity assessment was rated using binary ratings ('yes'/'no'), with items having clarity concerns accompanied by detailed expert feedback identifying specific issues.

The content validity assessment demonstrated excellent results across all measures. High inter-rater agreement (S-CVI/Ave > 0.9) in the first round eliminated the need for a second validation round, consistent with established methodology when initial agreement exceeds 0.90. Of the original 28 items:

- 24 items demonstrated acceptable relevance (I-CVI scores 0.75-1.0)
- 12 items achieved perfect relevance scores (I-CVI = 1.0)
- 10 items scored 0.87
- 1 item scored 0.85
- 4 items fell below the I-CVI threshold of 0.78 and were eliminated:
  - Item 16: 'on this ward the nurses make time to connect with me'
  - Item 17: 'on this ward the nurses connect with me on a personal level that I feel comfortable with'
  - Item 20: 'on this ward the nurses make me feel supported at a time when I am under stress'
  - Item 23: 'on this ward the nurses support me with dealing with challenges or problems that happen in relation to patient care'

Expert recommendations included:

- 17 items were rated as requiring no or minimal revisions
- 11 items were recommended for major revisions

Expert consensus on item clarity varied:

- 6 items: Unanimous clarity agreement (100%)
- 12 items: Strong consensus agreement (86%)
- 9 items: Moderate agreement (71%)
- 1 item: Rated as unclear by many experts (67%)

Through this comprehensive validation process, the original 28-item instrument was systematically refined to a 24-item scale. The development incorporated I-CVI relevance analysis, modified I-CVI recommendations, item clarity assessment, and expert qualitative feedback. The resulting 24-item instrument demonstrated excellent content validity (S-CVI/Ave = 0.93) and was ready for subsequent validation phases.

Reference

1. Polit D, Beck C. Nursing research: generating and assessing evidence for nursing practice. Philapdelphia. Wolters Kluwer. Available from: <https://ebookcentral>. proquest. com/lib …; 2021.

# Supporting file S4

Response process evidence (cognitive interview) protocol

*Framework*

Response process evaluation used cognitive interviewing with end users to assess comprehension, alignment with the construct definition, response option functioning, and acceptability, consistent with the Standards for Educational and Psychological Testing.^1^ This updates Boateng et al.’s terminology while retaining their sequential framework.^2^ This approach provides validity evidence by examining how respondents interpret and respond to survey items, moving beyond surface-level face validity to deeper assessment of cognitive processes. The protocol was informed by the Cognitive Interviewing Reporting Framework (CIRF)^3^ which provides a 10-point structured approach for testing, developing, and evaluating survey questions with the target population.^4^

*Methodology*

Cognitive interviewing methodology involves administering a draft survey while asking respondents to verbalise the mental processes involved in providing their answers. The purpose is to understand how respondents interpret and answer survey questions, allowing for modifications, clarifications, or augmentation to align with study objectives.^4^ Target population members are considered experts at evaluating face validity, making cognitive interviews with this group essential for ensuring items are meaningful to intended end-users before final administration.

The study used a hybrid approach combining think-aloud techniques -where participants verbalised their thoughts while answering questions and concurrent verbal probing where targeted questions were asked during item response.^3,5^ The hybrid approach use of both scripted and unscripted probes, allowed the interviewer to deviate when appropriate while maintaining consistency through standardised core questions. This facilitated thorough evaluation before instrument finalisation.

The cognitive interview protocol was designed to assess three components: 1) participant understanding of individual items, 2) construct alignment between participant interpretation and researcher intent, and 3) participant ability to appropriately use items and corresponding response categories.^3^ Additional evaluation criteria included respondent burden and overall acceptability of the instrument.

*Sample and setting*

While the literature recommends 10-20 participants ^6^ or 5-15 interviews across 2-3 rounds until saturation,^2^ this study adopted a pragmatic approach determined by available resources.^7^ A convenience sample of six participants representative of the target population ^6^ were recruited through personal and professional networks. All participants had experience of having an ill family member hospitalised. None were healthcare professionals to ensure independence from item development and capture the intended survey demographic.^2^ Four participants were based in South-East Queensland and interviewed in person while participants from North Queensland and Sydney were interviewed via Teams and telephone respectively.

**Table 1** *Participant demographics*

**
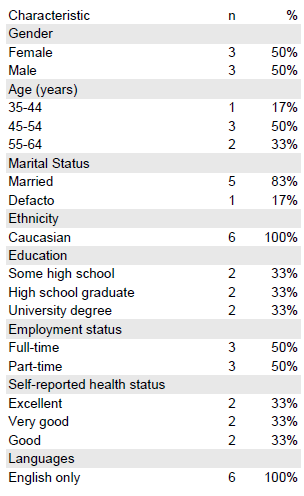
**

*Data collection*

A single Nurse Researcher interviewer with qualitative experience conducted all cognitive interviews to ensure consistency. All interviews lasted 20-25 minutes and were audio-recorded with supplementary notes taken during sessions.

The draft instrument included 24 items formatted following visual layout recommendations for instrument development,^8^ a five-point agreement response scale and a comments section for additional feedback. Following each interview, contact summaries were completed while listening to recordings to document key points and observations. Information power was achieved ^9^ after six Australian interviews, with no new information emerging and no adjustments to research protocol required. Demographic data were collected from all participants.

*Data analysis*

Inductive content analysis was undertaken following the procedures outlined in the CIRF.^3^ Data reduction included the transformation of individual comments from separate survey items across multiple interviews into a coherent set of summary findings.^3^ Original comments from the interviews were reviewed by all investigators during group meetings to inform analysis and interpretation of the findings. The interview process was considered reliable and systematic as only one researcher conducted all interviews. Demographic data was analysed using descriptive statistics to characterise the sample. Due to the small sample size, no computer software programs were required for analysis.

*Results*

Inductive content analysis of the cognitive interviews revealed several key recommendations for instrument refinement. Revisions included removing one repetitive item, consideration of providing a broader definition of 'family', reordering for cognitive flow, rewording specific items to enhance understanding and changing from agreement to frequency response option format. All participants agreed that the instructions were clear and easy to understand.

Following the cognitive interviews, a group meeting was held between the Australian and Dutch study teams to discuss the findings and finalise instrument modifications. The final version of the instrument, incorporating the recommended changes, comprised 23 items (reduced from 24).

References

1. Plake BS, Wise LL. What is the role and importance of the revised AERA, APA, NCME Standards for Educational and Psychological Testing? Educational Measurement: Issues and Practice. 2014;33(4):4-12.

2. Boateng GO, Neilands TB, Frongillo EA, Melgar-Quiñonez HR, Young SL. Frontiers in Public Health. 2018;6.

3. Boeije H, Willis G. The cognitive interviewing reporting framework (CIRF). Methodology. 2013.

4. Rodrigues IB, Adachi JD, Beattie KA, MacDermid JC. Development and validation of a new tool to measure the facilitators, barriers and preferences to exercise in people with osteoporosis. BMC Musculoskeletal disorders. 2017;18:1-9.

5. Willis GB. Analysis of the cognitive interview in questionnaire design: Oxford University Press; 2015.

6. Polit-O'Hara D, Beck CT. Resource manual for Nursing research : generating and assessing evidence for nursing practice. Ninth edition. ed. Philadelphia: Wolters Kluwer/Lippincott Williams & Wilkins; 2012.

7. Guest G, Bunce A, Johnson L. How many interviews are enough? An experiment with data saturation and variability. Field methods. 2006;18(1):59-82.

8. Pett MA, Lackey NR, Sullivan JJ. Making sense of factor analysis: The use of factor analysis for instrument development in health care research: sage; 2003.

9. Malterud K, Siersma VD, Guassora AD. Sample size in qualitative interview studies: guided by information power. Qualitative health research. 2016;26(13):1753-60.

# Supporting file S5

Inpatient unit specialities

Inpatient units included cardiology; cardiothoracic; urology/gynaecology/head and neck; neurosurgical/trauma; orthopaedics; cardiothoracic/trauma; renal; vascular/medical; gastrointestinal; respiratory; general medical; immunology; neurology and haematology/oncology; surgical; and acute medical. Both hospitals had established consumer engagement frameworks aligned with the Australian National Safety and Quality Health Service (NSQHS) Standard 2 (Partnering with Consumers),^1^ including patient safety campaigns such as Ryan's Rule, ^2^ Speak Up for Patient Safety (SUPFS),^2^ and consumer engagement procedure documents to guide staff. The study was presented to the Consumer Advisory Group (CAG) where consumer representatives confirmed the need for a standardised measure of family engagement and provided input on implementation considerations.

References

1. ACSQHC. Australian Commission on Safety and Quality in Health Care Person-centred care ACSQHC: Sydney2022 [Available from: <https://www.safetyandquality.gov.au/our-work/partnering-consumers/person-centred-care>

2. Government Q. Patient safety staff escalation. In: Queensland CE, editor. Brisbane: Queensland Government.

# Supporting file S6

The TOGETHER scale

INSTRUCTIONS FOR FAMILY MEMBERS:

About this survey:

Including family members in care is important because it can improve care quality. While providing safe high-quality care is the responsibility of all healthcare professionals, nurses play an important role in supporting families in the care experience. Nurses understand how important the family unit is and aim to create a supportive environment for the patient and their family.

By ‘family member’, we use a broad definition of family including both relatives and close friends that know the patient and either live with or are involved in the ongoing care of the patient. They are people who provide support and with whom the patient has a significant relationship.

**In this survey we would like your opinion of how nurses support you as a family member when you have a relative (the patient) in the hospital.** The survey should take around 10 minutes to complete. For simplicity we refer to your relative in this survey as ‘the patient’.

By participating in this survey, you can tell us about your experience and give us your opinion if you want to.

There are no right or wrong answers. Please answer all questions by selecting the best response.

**Before you start:**

Remember when choosing your answers to think about your experience of being **on this ward for this admission only** and no other areas of the hospital or other hospital admissions.

Let’s begin.

**The purpose of this survey is to explore your view of how nurses support you (the family**

**member) when you have a patient in the hospital.**

Please tell us how much you agree with each statement and leave comments if you would like to.

| Statements:  ↓ | Never | | Rarely | | Some-times | Often | | | Always | N/A or Comments: |
| --- | --- | --- | --- | --- | --- | --- | --- | --- | --- | --- |
| 1. The nurses make me feel that what I say is important | 1 | | 2 | | 3 | 4 | | | 5 |  |
| 2. The nurses encourage me to share information that I think is relevant with the healthcare team if I want to | 1 | | 2 | | 3 | 4 | | | 5 |  |
| 3. The nurses use the information that I provide to improve patient care | 1 | | 2 | | 3 | 4 | | | 5 |  |
| 4. The nurses give me information to help me understand what is happening | 1 | | 2 | | 3 | 4 | | | 5 |  |
| 5. The nurses provide updates without being asked | 1 | | 2 | | 3 | 4 | | | 5 |  |
| 6. The nurses ask if I am satisfied with the care provided | 1 | | 2 | | 3 | 4 | | | 5 |  |
| 7. The nurses ask me how I would like to be involved in caring for the patient | 1 | | 2 | | 3 | 4 | | | 5 |  |
| 8. The nurses support me to be involved in caring for the patient | 1 | | 2 | | 3 | 4 | | | 5 |  |
| 9.The nurses check that I feel comfortable when helping care for the patient | 1 | | 2 | | 3 | 4 | | | 5 |  |
| 10.The nurses ask me if I would like to be involved in making decisions about patient care | 1 | | 2 | | 3 | 4 | | | 5 |  |
| 11. The nurses make sure that I feel comfortable with being involved in decisions about patient care | 1 | | 2 | | 3 | 4 | | | 5 |  |
| 12. The nurses help me to understand the plan of care | 1 | 2 | | 3 | | | 4 | 5 | |  |
| Statements:  ↓ | Never | Rarely | | Some-times | | | Often | Always | | N/A or Comments: |
| 13. The nurses involve the patient and I in planning care and setting goals | 1 | 2 | | 3 | | | 4 | 5 | |  |
| 14. The nurses prioritise goals of care that are important to the patient and I | 1 | 2 | | 3 | | | 4 | 5 | |  |
| 15. The nurses make me feel that we are working together to help the patient | 1 | 2 | | 3 | | | 4 | 5 | |  |
| 16. The nurses talk to me in a respectful way | 1 | 2 | | 3 | | | 4 | 5 | |  |
| 17. The nurses talk to me in a way that is easy to understand | 1 | 2 | | 3 | | | 4 | 5 | |  |
| 18. I feel nurses answer my questions in an honest and accurate way | 1 | 2 | | 3 | | | 4 | 5 | |  |
| 19. I feel the nurses understand the effect the patient’s illness has on me | 1 | 2 | | 3 | | | 4 | 5 | |  |
| 20. The nurses allow me to visit according to my wishes | 1 | 2 | | 3 | | | 4 | 5 | |  |
| 21. The nurses respect my cultural needs | 1 | 2 | | 3 | | | 4 | 5 | |  |
| 22. The nurses respect my religious and/ or spiritual needs | 1 | 2 | | 3 | | | 4 | 5 | |  |
| 23. The nurses give me information to help me prepare for the patient’s discharge | 1 | 2 | | 3 | | | 4 | 5 | |  |

Please comment further if you would like to:

__________________________________________________________________________________________________________________________________________________

__________________________________________________________________________________________________________________________________________________

# Supporting File S7

Item-level descriptive statistics for the 20-item TOGETHER analytic set (N = 237)

| **Item** | **Item content (abbreviated)** | **Cat** | **n** | **Comp %** | **M** | **SD** | **Skewness** | **Kurtosis** |
| --- | --- | --- | --- | --- | --- | --- | --- | --- |
| Q1 | The nurses introduced themselves to me | 5 | 234 | 98.7 | 4.34 | 0.96 | -1.63 | 2.59 |
| Q2 | The nurses asked me about my role in caring for the patient | 5 | 222 | 93.7 | 3.99 | 1.28 | -1.08 | 0.04 |
| Q3 | The nurses asked me how I would like to be involved in the patient's care | 5 | 207 | 87.3 | 4.11 | 1.20 | -1.31 | 0.77 |
| Q4 | The nurses kept me informed about the patient's condition | 5 | 235 | 99.2 | 4.07 | 1.18 | -1.14 | 0.36 |
| Q5 | The nurses encouraged me to ask questions about the patient's care | 5 | 232 | 97.9 | 3.39 | 1.40 | -0.41 | -1.06 |
| Q6 | The nurses asked me if I was satisfied with the care provided to the patient | 5 | 223 | 94.1 | 2.45 | 1.57 | 0.55 | -1.26 |
| Q7 | The nurses asked me about my preferences for being involved in the patient's care | 5 | 217 | 91.6 | 2.25 | 1.52 | 0.83 | -0.86 |
| Q8 | The nurses supported me to be involved in the patient's care | 5 | 224 | 94.5 | 3.26 | 1.58 | -0.29 | -1.43 |
| Q9 | The nurses made me feel comfortable being involved in the patient's care | 5 | 211 | 89.0 | 3.11 | 1.64 | -0.14 | -1.60 |
| Q10 | The nurses invited me to participate in care planning or decisions about the patient's care | 5 | 207 | 87.3 | 2.65 | 1.66 | 0.34 | -1.55 |
| Q11 | The nurses made sure I was comfortable with my level of involvement and decision-making | 5 | 200 | 84.4 | 3.08 | 1.63 | -0.16 | -1.58 |
| Q12 | The nurses involved me in setting goals for the patient's care | 5 | 220 | 92.8 | 3.59 | 1.47 | -0.65 | -0.97 |
| Q13 | The nurses and I worked together to plan the patient's care | 5 | 206 | 86.9 | 3.25 | 1.57 | -0.29 | -1.44 |
| Q14 | The nurses involved me in decisions about the patient's care | 5 | 210 | 88.6 | 3.58 | 1.51 | -0.64 | -1.04 |
| Q15 | The nurses valued my input into the patient's care | 5 | 225 | 94.9 | 3.77 | 1.40 | -0.82 | -0.62 |
| Q16 | The nurses spoke to me with respect | 5 | 236 | 99.6 | 3.79 | 0.50 | -2.57 | 6.87 |
| Q17 | The nurses communicated with me in a way that was easy to understand | 5 | 235 | 99.2 | 3.71 | 0.58 | -2.01 | 3.55 |
| Q18 | The nurses treated the patient with respect | 5 | 233 | 98.3 | 4.60 | 0.80 | -2.32 | 5.52 |
| Q19 | The nurses respected the patient's wishes about their care | 5 | 215 | 90.7 | 3.91 | 1.29 | -1.03 | 0.00 |
| Q20 | The nurses allow me to visit according to my wishes | 5 | 220 | 92.8 | 3.64 | 0.76 | -2.10 | 3.49 |

***Note.*** *Cat = number of response categories; Comp % = completion rate (n / 237 × 100); M = mean; SD = standard deviation. Skewness and kurtosis are sample estimates with bias correction.*

# Supporting File S8

TOGETHER Scoring Guide

*Practical instructions for scoring and reporting the TOGETHER Family-Reported Experience Measure*

**Overview**

The TOGETHER scale comprises 23 items assessing family-reported experiences of nurse-facilitated engagement in adult acute care. For scoring purposes, items are classified into two groups:

**Core items (Q1–Q20):** These 20 items form the structurally analysed core of the instrument and are used to derive the TOGETHER total score.

**Supplementary items (Q21–Q23):** These three context-contingent items (cultural needs, religious/spiritual needs, discharge information) are retained for clinical coverage and equity but are scored and reported separately from the total score. They do not contribute to the TOGETHER total score.

**Item classification and factor assignment**

| **Item** | **Item content** | **Factor assignment** | **Scoring status** |
| --- | --- | --- | --- |
| **Q1** | The nurses introduced themselves to me | S1 (Communication) | Core |
| **Q2** | The nurses asked me about my role in caring for the patient | S1 (Communication) | Core |
| **Q3** | The nurses asked me how I would like to be involved in the patient’s care | S1 (Communication) | Core |
| **Q4** | The nurses kept me informed about the patient’s condition | S1 (Communication) | Core |
| **Q5** | The nurses encouraged me to ask questions about the patient’s care | S1 (Communication) | Core |
| **Q6** | The nurses asked me if I was satisfied with the care provided to the patient | General only | Core |
| **Q7** | The nurses asked me about my preferences for being involved in the patient’s care | General only | Core |
| **Q8** | The nurses supported me to be involved in the patient’s care | General only | Core |
| **Q9** | The nurses made me feel comfortable being involved in the patient’s care | General only | Core |
| **Q10** | The nurses invited me to participate in care planning or decisions about the patient’s care | General only | Core |
| **Q11** | The nurses made sure I was comfortable with my level of involvement and decision-making | General only | Core |
| **Q12** | The nurses involved me in setting goals for the patient’s care | S2 (Collaboration) | Core |
| **Q13** | The nurses and I worked together to plan the patient’s care | S2 (Collaboration) | Core |
| **Q14** | The nurses involved me in decisions about the patient’s care | S2 (Collaboration) | Core |
| **Q15** | The nurses valued my input into the patient’s care | S2 (Collaboration) | Core |
| **Q16** | The nurses spoke to me with respect | S1 (Communication) | Core |
| **Q17** | The nurses communicated with me in a way that was easy to understand | S1 (Communication) | Core |
| **Q18** | The nurses treated the patient with respect | S1 (Communication) | Core |
| **Q19** | The nurses respected the patient’s wishes about their care | S1 (Communication) | Core |
| **Q20** | The nurses allow me to visit according to my wishes | S1 (Communication) | *Provisional†* |
| **Q21** | The nurses respected my cultural needs | — | *Supplement‡* |
| **Q22** | The nurses respected my religious or spiritual needs | — | *Supplement‡* |
| **Q23** | The nurses provided me with information about discharge planning | — | *Supplement‡* |

*† Q20 is provisionally retained to preserve content coverage of visitation practices. Structural performance was weak in the development sample (h² = 0.29, λg = 0.27) and requires re-evaluation in future validation studies. If weak performance persists, the item should be revised or removed.*

*‡ Q21–Q23 were excluded from factor modelling due to high context-contingent missingness (41–73%). Their retention is based on Phase 1 content-validity evidence (expert review, S-CVI/Ave = 0.93; cognitive interviewing) and clinical relevance rather than internal-structure evidence.*

**Scoring procedure for the TOGETHER total score**

**Step 1. Identify eligible responses.**

The total score is derived from the 20 core items (Q1–Q20) only. Items Q21–Q23 are excluded from the total score calculation.

**Step 2. Handle ‘not applicable’ responses.**

‘Not applicable’ responses should be treated as missing data, not recoded as zero. A ‘not applicable’ response indicates that the relevant care experience did not arise during the admission, which is not equivalent to a negative evaluation of engagement.

**Step 3. Apply the minimum completion threshold.**

A respondent must have completed at least 16 of the 20 core items (80%) for a total score to be calculated. If fewer than 16 items are completed, the case should be treated as having insufficient data for scoring. This threshold is a pragmatic scoring rule for this instrument, adopted to balance data inclusion against score reliability.

**Step 4. Calculate the total score.**

The TOGETHER total score is calculated as the mean of completed core items:

*Total score = sum of completed item responses / number of completed items*

Higher scores indicate more positive family-reported experiences of nurse-facilitated engagement. The mean-based scoring automatically adjusts for the number of items completed (provided the minimum threshold is met).

**Step 5 (optional). Transform the score if required.**

Two alternative formats are available for settings where they are useful:

| **Scoring method** | **Formula** | **Example (sum = 68, items completed = 18)** |
| --- | --- | --- |
| Mean score | Sum of responses / number of completed items | 68 / 18 = 3.78 |
| Prorated total | Mean score × 20 | 3.78 × 20 = 75.56 |
| 0–100 transformation | ((Mean score – 1) / (max – 1)) × 100 | ((3.78 – 1) / (4)) × 100 = 69.44 |

***Note.*** *The 0–100 transformation assumes a response scale ranging from 1 to 5 for the majority of items. Where items use fewer response categories, users should verify that the maximum possible value (max) is appropriate for their version.*

**Subscale and domain-level reporting**

The bifactor analysis identified two specific factors: S1 (Communication: Q1–Q5, Q16–Q20) and S2 (Collaboration: Q12–Q15). Items Q6–Q11 loaded on the general factor only.

**Important:** The specific factors retained limited unique reliable variance beyond the general factor (ωhs communication = 0.16; ωhs collaboration = 0.04). Subscale scores therefore lack sufficient reliability for standalone use in confirmatory hypothesis testing, clinical decision-making, or between-group comparisons. Domain-level item means may be examined descriptively (e.g., to identify areas of relative strength or weakness within a service) but should not be treated as independent psychometric outcomes.

**Reporting supplementary items (Q21–Q23)**

Q21 (cultural needs), Q22 (religious/spiritual needs), and Q23 (discharge information) should be reported separately at the item level. The following guidance applies:

- **Do not include in the total score.** These items are not part of the scored latent core.
- **Report item-level frequencies and means.** Descriptive statistics (e.g., response distributions, completion rates) provide useful targeted feedback regarding culturally responsive care, spiritual support, and discharge communication.
- **Treat ‘not applicable’ as structural missingness.** A ‘not applicable’ response indicates the care situation did not arise, not that the respondent had a negative experience. Analysts should pre-specify how these responses will be handled before data collection.
- **Interpret completion rates as service-level indicators.** A high proportion of ‘not applicable’ responses may indicate that cultural assessment, spiritual support, or discharge-planning conversations are not routinely offered, rather than that families do not value them.

**Summary of scoring recommendations**

| **Component** | **Recommendation** |
| --- | --- |
| **Total score (Q1–Q20)** | Mean of completed core items. Minimum 16/20 items required. Primary outcome for evaluation and monitoring. |
| **Subscale scores** | May be examined descriptively. Insufficient reliability for standalone confirmatory use. |
| **Q20 (visitation)** | Included in total score but provisionally retained. Re-evaluate in future validation samples. |
| **Q21–Q23 (supplements)** | Report separately at item level. Do not include in total score. Treat ‘not applicable’ as structural missingness. |
| **‘Not applicable’ responses** | Treat as missing (not zero) for all items. |
| **Cross-group comparison** | Not recommended until measurement invariance is established in future studies. |
